# Fo Shou San, an Ancient Chinese Herbal Decoction, Protects Endothelial Function through Increasing Endothelial Nitric Oxide Synthase Activity

**DOI:** 10.1371/journal.pone.0051670

**Published:** 2012-12-21

**Authors:** Cathy W. C. Bi, Li Xu, Xiao Yu Tian, Jian Liu, Ken Y. Z. Zheng, Chi Wai Lau, David T. W. Lau, Roy C. Y. Choi, Tina T. X. Dong, Yu Huang, Karl W. K. Tsim

**Affiliations:** 1 Division of Life Science and Center for Chinese Medicine, The Hong Kong University of Science and Technology, Hong Kong, China; 2 Institute of Vascular Medicine, Li Ka Shing Institute of Health Sciences, School of Biomedical Sciences, Chinese University of Hong Kong, Hong Kong, China; Medical College of Wisconsin, United States of America

## Abstract

Fo Shou San (FSS) is an ancient herbal decoction comprised of Chuanxiong Rhizoma (CR; Chuanxiong) and Angelicae Sinensis Radix (ASR; Danggui) in a ratio of 2∶3. Previous studies indicate that FSS promotes blood circulation and dissipates blood stasis, thus which is being used widely to treat vascular diseases. Here, we aim to determine the cellular mechanism for the vascular benefit of FSS. The treatment of FSS reversed homocysteine-induced impairment of acetylcholine (ACh)-evoked endothelium-dependent relaxation in aortic rings, isolated from rats. Like radical oxygen species (ROS) scavenger tempol, FSS attenuated homocysteine-stimulated ROS generation in cultured human umbilical vein endothelial cells (HUVECs), and it also stimulated the production of nitric oxide (NO) as measured by fluorescence dye and biochemical assay. In addition, the phosphorylation levels of both Akt kinase and endothelial NO synthases (eNOS) were markedly increased by FSS treatment, which was abolished by an Akt inhibitor triciribine. Likewise, triciribine reversed FSS-induced NO production in HUVECs. Finally, FSS elevated intracellular Ca^2+^ levels in HUVECs, and the Ca^2+^ chelator BAPTA-AM inhibited the FSS-stimulated eNOS phosphorylation. The present results show that this ancient herbal decoction benefits endothelial function through increased activity of Akt kinase and eNOS; this effect is causally via a rise of intracellular Ca^2+^ and a reduction of ROS.

## Introduction

Fo Shou San (FSS) composed of Angelicae Sinensis Radix [ASR; Danggui; the roots of *Angelica sinensis* (Oliv) Diels.] and Chuanxiong Rhizoma (CR; Chuanxiong; the rhizomes of *Ligusticum chuanxiong* Hort.) in a weight ratio of 3∶2, is an ancient Chinese herbal decoction. The first description of FSS was recorded by *Xu Shuwei* in *Puji Benshi* in Song Dynasty (AD 1132) of China, and indeed this decoction was being used clinically for hundreds of years. According to traditional Chinese medicine (TCM) theory and practice, FSS should be functioned in “Nourishing Blood” and “Promoting Blood Circulation”. Historically, FSS was originally prescribed for treating women aliments, especially for those suffering obstetric diseases, such as dystocia, vaginal bleeding with fetal movement, dead fetus in uterus and post-partum anemic fainting. Our recent reports suggested that FSS was capable of inducing the differentiation of erythropoietic precursor cells and the expression of erythropoietin (EPO), an erythrocyte-specific hematopoietic growth factor, in cultured liver cells [1]. While, another major property of FSS in promoting blood circulation was testified by its ability to inhibit ADP-induced platelet aggregation [2].

The endothelium is a thin layer of cells covering the interior surface of blood vessels, as to form an interface between circulating blood in the lumen and the rest of vascular wall. The best characterized endothelium-derived relaxing factor (EDRF), nitric oxide (NO) dilates blood vessels, increases blood flow, and inhibits platelet activation and angiogenesis [3]. The production of NO, triggered by endothelial nitric oxide synthases (eNOS, endothelial isoform of NO synthases), in endothelial cells has been well studied. The activation of eNOS could be achieved by its phosphorylation at Ser 1177, and this eNOS phosphorylation can be triggered by the activation of protein kinase Akt (also known as protein kinase B) and phosphoinositide-3-kinase (PI3 kinase) [4]. An important mode of inactivation of NO is its reaction with superoxide anion (O_2_•^−^) to form the potent oxidant peroxynitrite (ONOO_2_•^−^) [5], [6]. A functional eNOS protein is a homodimer, which transfers electrons from NADPH, via the flavins FAD and FMN in the carboxyterminal reductase domain, to the haem in the amino-terminal oxygenase domain [7], [8]. The majority of ROS generation in the vasculature is derived from NADPH oxidases and eNOS uncoupling. The latter takes place when oxidative stress oxidizes the fragile eNOS cofactor tetrahydrobiopterin (BH4) [9]. Evidences have shown that eNOS uncoupling is one of the underlying causes of endothelial dysfunction in animal experiments [10], [11], [12], [13], [14], [15]. Moreover, the activation of eNOS also depends on the binding of ubiquitous intracellular calcium regulatory protein, calmodulin [16]. In order to demonstrate the “Promoting Blood Circulation” property of FSS, the present study investigated whether FSS protected endothelial function, if so, the underlying mechanisms in relation to NO production and ROS reduction in endothelial cells.

## Results

### Preparation of Standardized FSS

To standardize FSS chemically, a typical HPLC fingerprint of FSS at an absorbance of 280 nm was developed **(Figure S1)**: this fingerprint served a purpose of herbal identification. By determining the amounts of ferulic acid and Z-ligustilide in the decoction, we were able to standardize FSS for the subsequent studies on their biological activities described below. A standardized FSS should contain no less than 120±7 mg of ferulic acid and 30±6 mg of Z-ligustilide in 100 g of the dry herbs. The extracting yield of FSS was about 45±5% (*n* = 3). These parameters established the chemical standards of FSS, as reported previously [2]: a standardized FSS was a prerequisite to perform the below biochemical analyses.

### FSS Protects Endothelial Function Against Homocysteine

**Figure 1:**
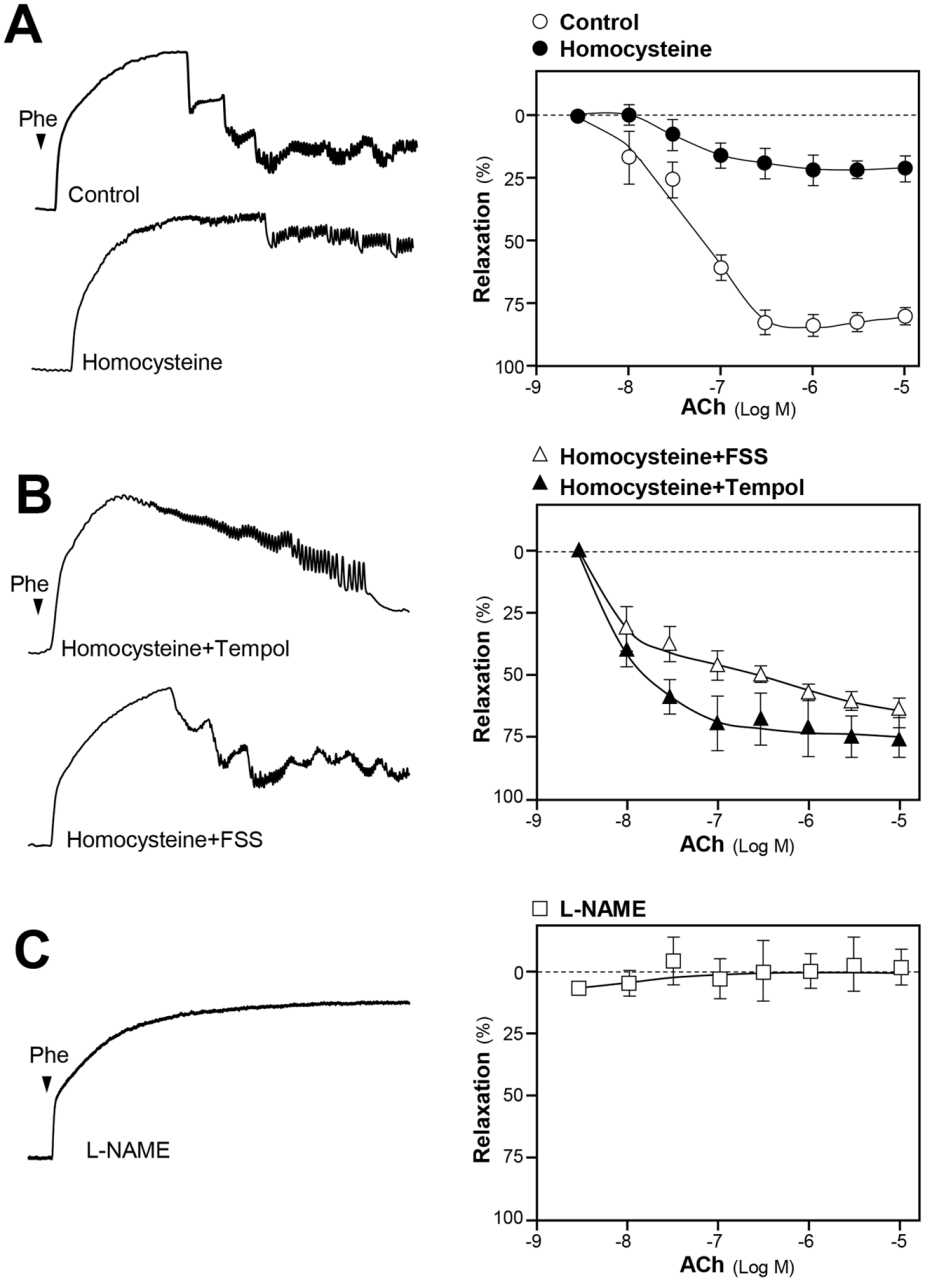
FSS protects homocysteine-induced endothelial dysfunction. (**A**): Rat aortic ring was isolated. The tension responsible for the vascular relaxation and constriction was tested after the treatment of different drugs. The contraction was induced by 0.5 µM phenylephrine (Phe). The endothelium dysfunction was induced by homocysteine (300 µM, a ROS inducer) for 60 min. The ring was suspended between two stainless steelwires in a 10-ml chamber on a Multi Myograph, which was used to measure the tension. Concentration-response curves (right panel) for acetylcholine (ACh from 0.01 to 10 µM) in the absence (control), or in the presence, of homocysteine. (**B**): The contraction of aortic ring was tested similar to (**A**). FSS (3 mg/ml) and tempol (1 µM; a ROS scavenger) were co-treated after the aorta rings were applied with homocysteine (300 µM) for 60 min in ACh-induced aortic relaxation. (**C**): The contraction of aortic ring was tested similar to (**A**). L-NAME (100 µM) was pre-treated in ACh-induced aortic relaxation. Values are expressed as percentage of relaxation as comparing to the control resting tension. Mean ± SEM, *n* = 3.

Endothelial dysfunction refers mainly to the reduced NO bioavailability or/and the over-produced radical oxygen species (ROS) [17]. Oxidative stress or increased ROS generation are implicated in hyper-homocysteinemia [18], which is a common and independent risk factor for atherosclerosis and other cardiovascular diseases. In an isolated aortic ring, acetylcholine (ACh) was shown to induce endothelium-dependent relaxations, and the relaxations were abolished by NOS inhibitor L-NAME [19]. Incubation of rat aortic rings with 300 µM homocysteine for 60 min markedly attenuated the ACh-induced relaxations **(** **Figure 1A** **)**, suggesting that homocysteine was effective in impairing endothelial function. Pre-treatment with FSS for 30 min after the challenge of homocysteine, the ACh-induced relaxations were largely restored **(** **Figure 1B** **)**, i.e. showing a vaso-protective effect of FSS. The effect of homocysteine in aortic ring was mainly mediated by the formation of ROS, because different ROS inhibitors could regulate the effect (**Figure S2**). Tempol, a ROS scavenger, served as a positive control. In addition, the application of L-NAME attenuated the ACh-induced vasodilation (**Figure 1C**), which suggested that ACh-induced vascular relaxation could be endothelial dependent. Similar to ACh effect, the role of FSS in aortic ring relaxation required endothelium, because the effect could not be revealed in the absence of endothelium (**Figure S3**). The time-dependent internal control of rat aortic ring relaxation to homocysteine alone, or the co-treatment of homocysteine with FSS, tempol, apocynin and catalase were tested without being induced by the application of ACh **(Figure S4)**.

### FSS Inhibits Homocysteine-stimulated ROS Production in Cultured HUVECs

**Figure 2:**
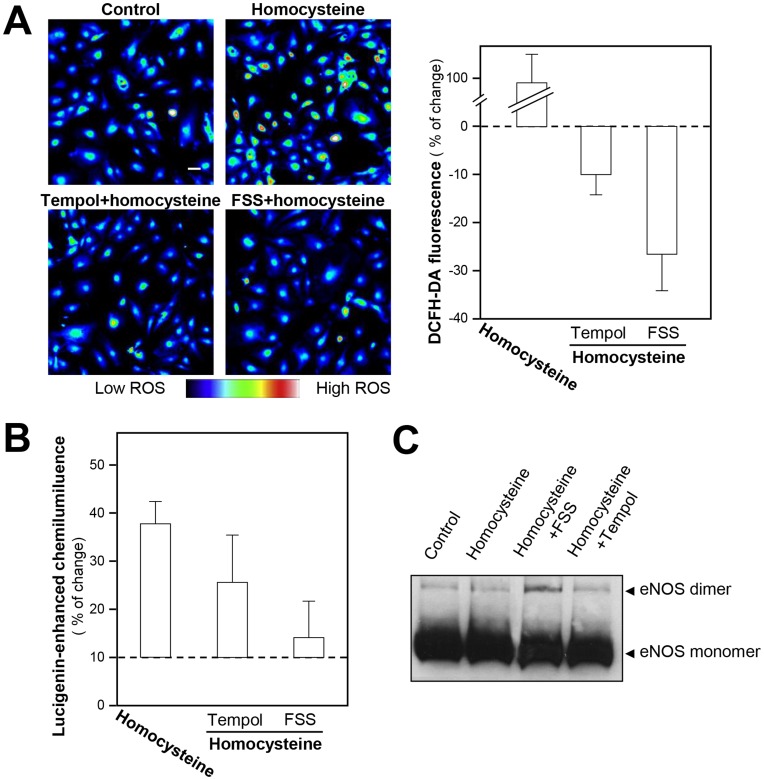
FSS blocks homocysteine-induced ROS formation. (**A**): Cultured HUVECs were pre-treated with FSS (1 mg/ml), or tempol (1 µM), or control (without drug treatment) for 30 min, and then add homocysteine (300 µM, a ROS inducer) for 30 min to induce the ROS formation. The cells were then labeled with DCFH-DA (1 µM) for 30 min, then the cells were washed with 1× NPSS (pH = 7.4) and the photos were taken by using laser confocal fluorescent microscopy, which the fluorescence intensity excited at 505 nm and emitted at 535 nm (left panel). Quantitation of fluorescent was calculated from the labeled HUVECs (right panel). Values are expressed as the percentage of change in the intensity of fluorescent, as compared to control cultures. Mean ± SEM, *n* = 3. (**B**): Cultured HUVECs were seeded in cell culture flask. The cultures were pre-treated with homocysteine (300 µM) for 30 min. The cells were then treated with FSS (1 mg/ml), or tempol (1 µM, positive control), or control (without drug treatment) for another 30 min. The cells in a concentration of 5×10^4^ per well on a 96-well white plate were then subjected to chemiluminescence determination. Values are expressed as percentage of increase to basal reading (control group). Mean ± SEM, *n* = 4. (**C**): Cultured HUVECs were seeded in 6-well plates. The cultures were pre-treated with homocysteine (300 µM) for 30 min. The cells were then treated with FSS (1 mg/ml), or tempol (1 µM, positive control), or control (without drug treatment) for another 30 min. eNOS protein was revealed by specific antibodies. The amount of eNOS dimer was increased by the application of FSS, or tempol, in homocysteine-treated cells.

A strong correlation between plasma homocysteine level and ROS production was observed in patients with cardiovascular diseases [20], [21]. Therefore, the suppression of ROS could be a potential therapeutic strategy in preventing or retarding endothelial dysfunction. Human umbilical vein endothelial cell (HUVEC) is a commonly used cell line for investigating the vascular pathology. Here, the application of homocysteine at 300 µM induced a significant rise of ROS formation in cultured HUVECs **(** **Figure 2** **)**. Tempol, a ROS scavenger, used as a positive control, inhibited homocysteine-stimulated ROS formation. Application of FSS reversed homocysteine-induced ROS production in cultured HUVECs **(** **Figure 2A** **)**, and this inhibition was stronger than that caused by tempol **(** **Figure 2A** **)**. Besides, the treatment of FSS, or tempol, also suppressed the basal level of ROS in HUVECs. The vaso-protective effect of FSS in cultured HUVECs was revealed by the detection of lucigenin-enhanced chemiluminescence (**Figure 2B**), a method similar to DCFH staining method. Oxidative stress has been shown to convert eNOS from a NO-producing enzyme to an enzyme that generates superoxide, a process termed NOS uncoupling (the dissociation of eNOS dimer). Therefore, the homocysteine-induced eNOS uncoupling model was involved. In addition, homocysteine induced the uncoupling of eNOS **(** **Figure 2C** **)**, which could be rescued by the application of FSS, or tempol **(** **Figure 2C** **)**. These results suggested that FSS could protect this homocysteine-caused endothelial dysfunction. The application of homocysteine significantly blocked the FSS-induced eNOS phosphorylation while partially blocked the VEGF-mediated eNOS phosphorylation **(Figure S5)**.

### FSS Increases NO Production in HUVECs

The vascular endothelium is the primary source for NO production in response to chemical or physical stimuli in blood vessel. Here, we employed HUVECs for the measurement of NO production. Cultured HUVECs were treated with FSS, and MTT assay was performed to assess cell viability. FSS treatment did not change the cell morphology (data not shown), but the MTT results showed that 1 mg/ml FSS, the commonly used concentration here, increased the proliferation of HUVECs by ∼50% **(Figure S6)**.

**Figure 3:**
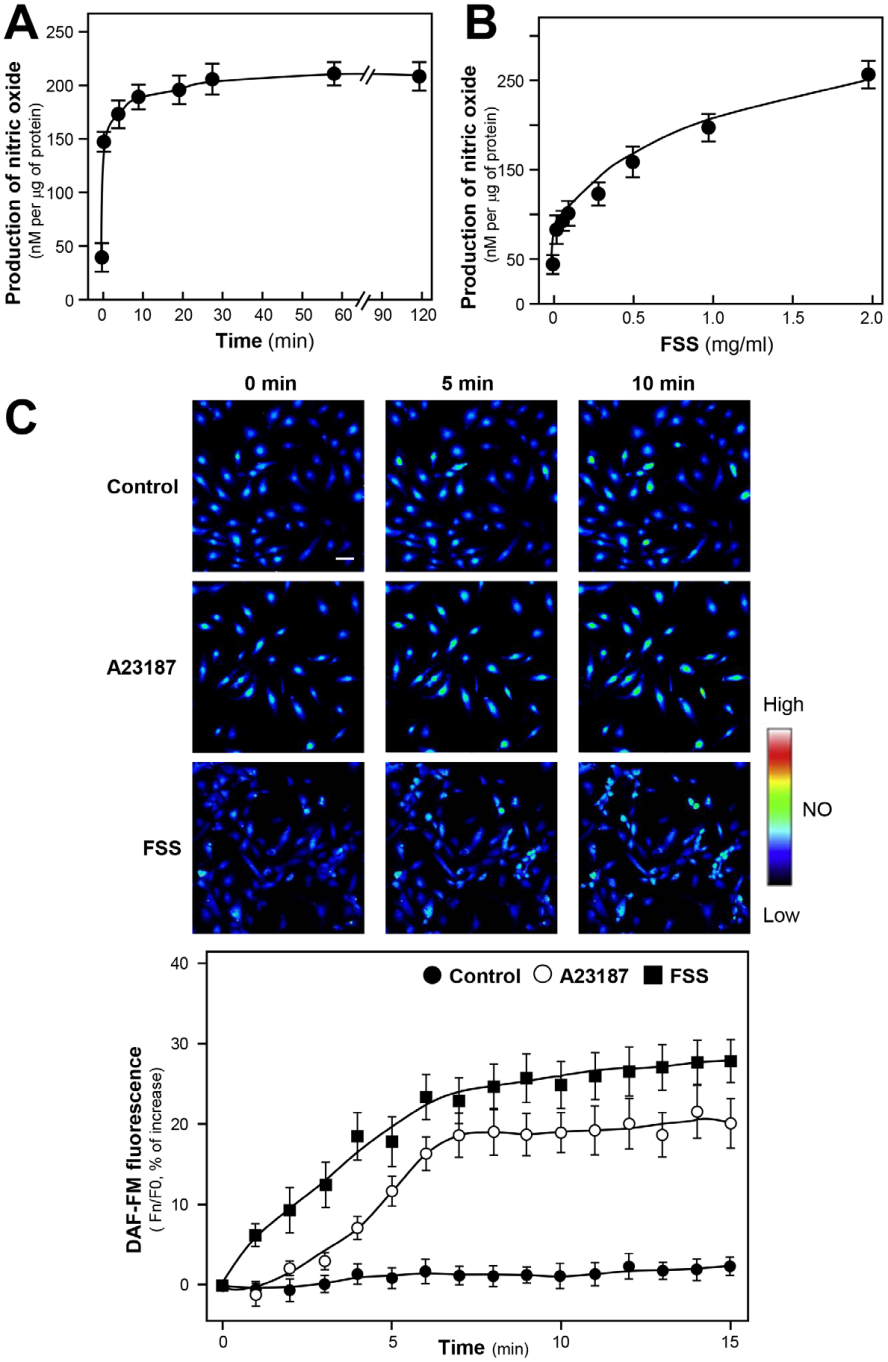
FSS stimulates the production of NO in cultured HUVECs. (**A**): Cultured HUVECs were treated with FSS (1 mg/ml) at different time point (0–120 min). (**B**): The cultures were treated with FSS (0–2 mg/ml) for 30 min. Total NO production was calculated from a calibration curve generated from standards provided by the manufacturer and normalized by the protein content of the corresponding well. Data are expressed as nM of NO production in 1 µg of protein. (**C**): Cultured HUVECs were labeled with fluorescent NO indicator DAF-FM DA for 30 min. Then the cells were washed with 1× NPSS (pH = 7.4), and then fluorimetric measurements were performed after the treatment of FSS (1 mg/ml), A23187 (1 µM, positive control), or control (without drug treatment). The amount of NO was evaluated by measuring the fluorescence intensity excited at 495 nm and emitted at 515 nm. Micrographs were taken by the confocal microscope (upper panel). Quantitation of intracellular NO production was displayed as a ratio of fluorescence intensity at any time (Fn) to the control at time 0 (F0) in the cultures (lower panel). Mean±SEM, *n* = 3. Bar = 50 µm.

**Figure 4:**
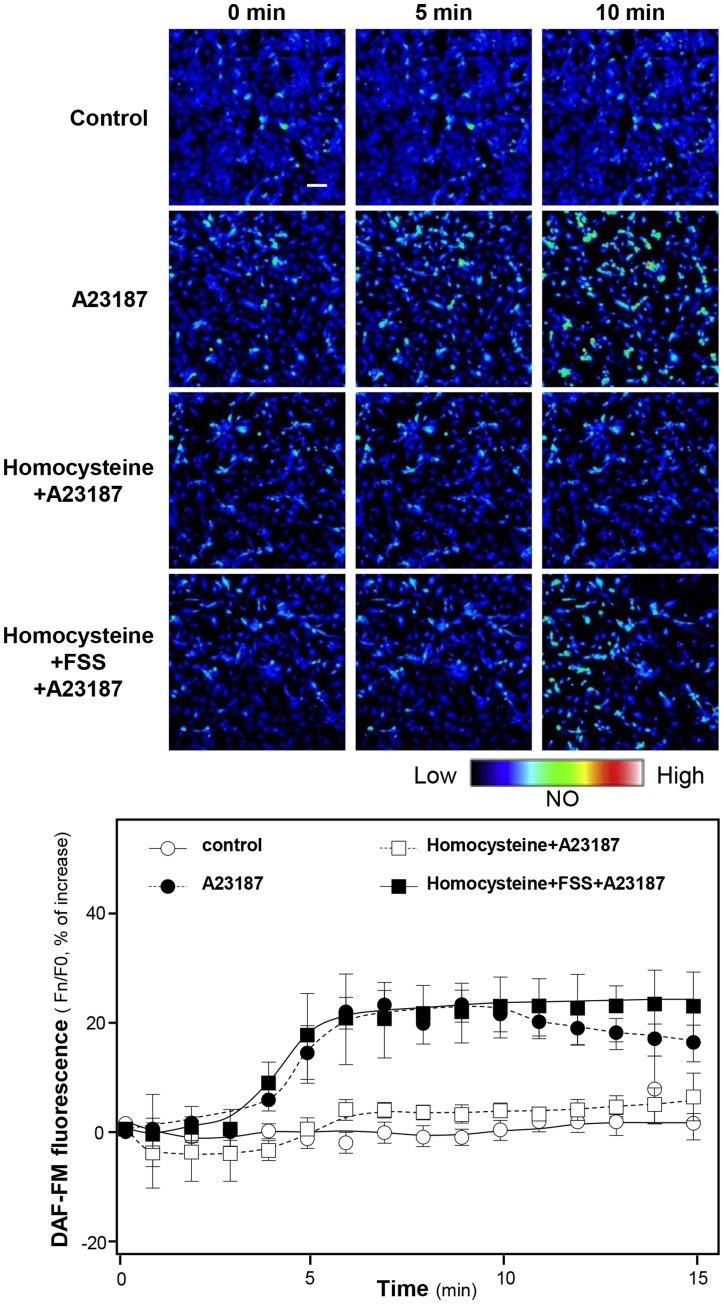
FSS rescues homocysteine-caused the decrease of NO production in cultured HUVECs after the application of A23187. Cultured HUVECs were labeled with fluorescent NO indicator DAF-FM DA for 30 min. Then, the cells were washed with 1× NPSS (pH = 7.4), and then fluorimetric measurements were performed after the treatment of A23187 (1 µM, positive control), or control (without drug treatment). For the pre-treatment, the cells were pretreated with homocysteine (300 µM) for 30 min, or with homocysteine (30 min) then added FSS (1 mg/ml) for another 30 min. The treatment of homocysteine did not affect the NO production (data not shown). After that, the cells were observed under confocal after the treatment of A23187 (1 µM). The amount of NO was evaluated by measuring the fluorescence intensity excited at 495 nm and emitted at 515 nm. Micrographs were taken by the confocal microscope (upper panel). Values are expressed as percentage of relaxation as comparing to the control resting tension (lower panel). Mean ± SEM, *n* = 4. Bar = 50 µm.

The production of NO was determined by using Griess reaction assay in cultured HUVECs. Application of FSS (1 mg/ml) stimulated a time-dependent rise in the intracellular NO production, which reached a steady level at around 10 min **(** **Figure 3A** **)**. FSS also produced a dose-dependent stimulation of NO production in HUVECs **(** **Figure 3B** **)**. The stimulated NO production was confirmed by laser confocal fluorescent microscopy using a specific dye: 4-amino-5-methylamino-20, 70-difluorofluorescein diacetate (DAF-FM DA). Application of FSS triggered a progressive rise in intracellular NO production in cultured HUVECs, as reflected by the increase of fluorescence intensity, which peaked at around 10 min after FSS addition **(** **Figure 3C** **)**. A23187, a calcium ionophore, was employed as a positive control to evoke NO production **(** **Figure 3C** **)**. The present results strongly indicate that FSS could elevate the NO production in human endothelial cells. Since FSS could rescue the homocysteine-induced endothelial dysfunction, therefore, HUVECs were first challenged with homocysteine for 45 min to induce the endothelial dysfunction. **Figure 4** shows that A23187 could not obviously induce NO production in cultured HUVECs. After the treatment of homocysteine, the cells were applied with FSS for 30 min. After that, the treatment of A23187 could significantly induce NO production **(** **Figure 4** **)**. These results further suggested that FSS could protect endothelial dysfunction, which was caused by oxidative stress.

**Figure 5:**
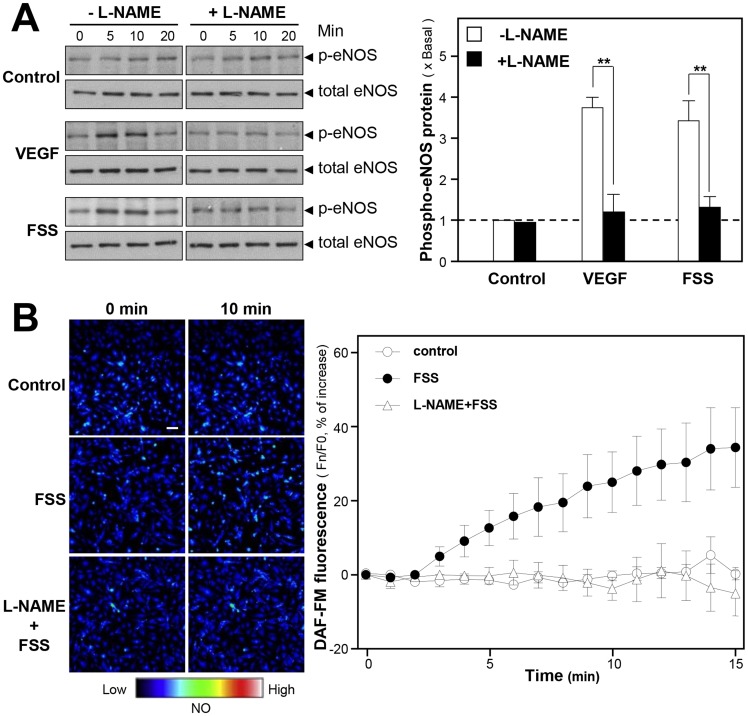
The FSS-induced NO production is mediated by eNOS phosphorylation at S1177. (**A**): Cultured HUVECs were seeded in 12-well plates. The cultures were serum starved for 3 hours before the pre-treatment with L-NAME (100 µM) for another 3 hours. The cells were then treated with FSS (1 mg/ml), or VEGF (20 ng/ml, positive control), or control (without drug treatment), at different time points. Total and phosphorylated eNOS was revealed by using specific antibodies (left panel). The quantification from the blots was shown by a densitometer (right panel). Data are expressed as × Basal where control was set as 1, Mean±SEM, *n = 4*. ** p<0.01. (**B**): Cultured HUVECs were labeled with fluorescent NO indicator DAF-FM DA for 30 min. Then, the cells were washed with 1× NPSS (pH = 7.4), and then fluorimetric measurements were performed after the treatment of FSS (1 mg/ml), or control (without drug treatment). For the pre-treatment of L-NAME, the cells were pre-treated with L-NAME (100 µM, eNOS blocker) for 30 min, after labeling with DAF-FM DA, NO production was induced by FSS (1 mg/ml). The amount of NO was evaluated by measuring the fluorescence intensity excited at 495 nm and emitted at 515 nm. Micrographs were taken by the confocal microscope (left panel). Values are expressed as percentage of relaxation as comparing to the control resting tension (right panel). Mean ± SEM, *n* = 4. Bar = 50 µm.

To investigate the possible role of eNOS and its upstream regulators, the phosphorylation level of eNOS was first determined to reflect the eNOS activity. The phosphorylation of eNOS at S1177 (at ∼135 kDa) in HUVECs was increased by a 5-min treatment of 1 mg/ml FSS **(** **Figure 5A** **)**. Vascular endothelial growth factor (VEGF), serving as positive control, robustly elevated eNOS phosphorylation. Either FSS-induced or VEGF-induced eNOS phosphorylation was fully abolished by 100 µM L-NAME, a NOS inhibitor **(** **Figure 5A** **)**. Likewise, L-NAME abolished partially the FSS-stimulated NO production in HUVECs **(** **Figure 5B** **)**, thus suggesting that the FSS-induced NO production was mediated by eNOS. The downstream effector of eNOS, cGMP, was regulated in parallel to the NO production in responding to different drug applications (**Figure S7**).

**Figure 6:**
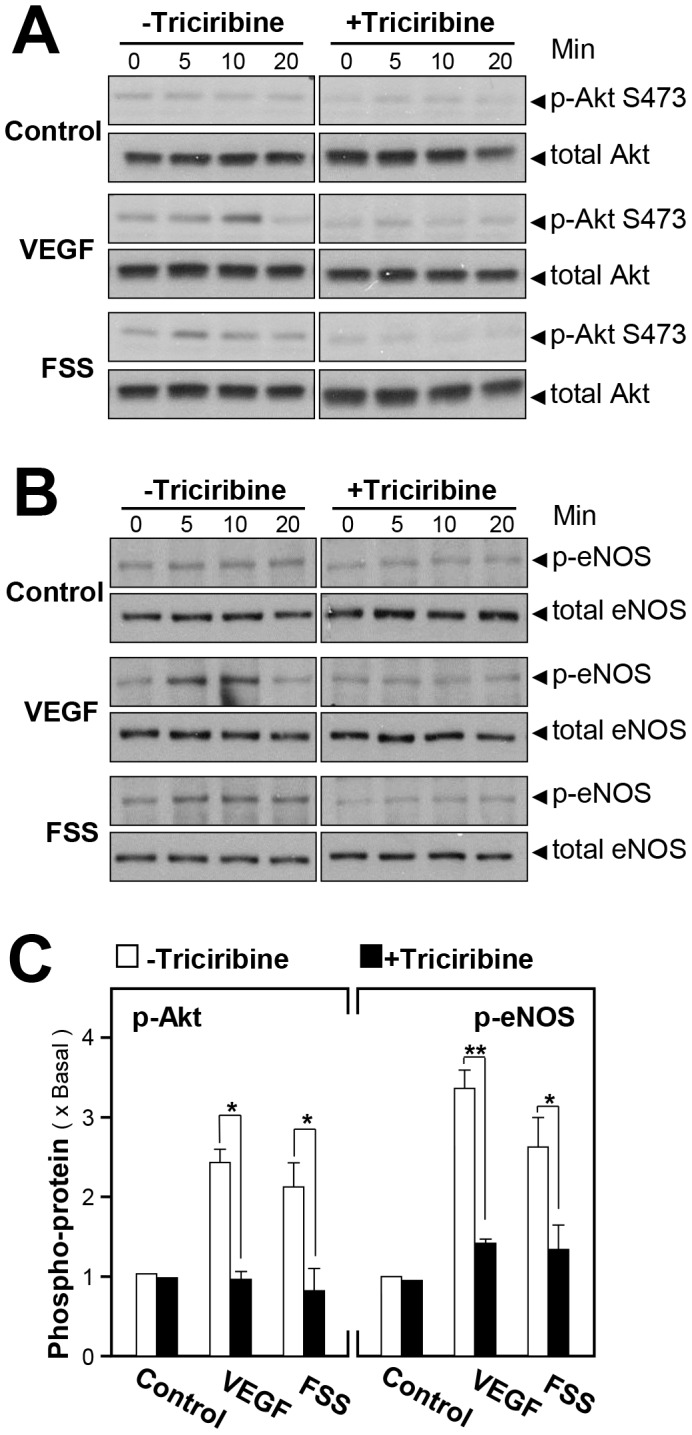
FSS-induced NO production is mediated by the phosphorylation of Akt at S473. Cultured HUVECs were pre-treated with triciribine (1 µM) for 3 hours before the application of FSS (1 mg/ml), or VEGF (20 ng/ml, positive control), or control (without drug treatment) for different time points. The cell lysates were obtained for western blotting. (**A**): Phospho-Akt S473 (∼60 kDa) and total Akt (∼60 kDa) were revealed by using specific antibodies of phospho-Akt S473 and total Akt. (**B**): Phospho-eNOS (∼135 kDa) and total eNOS (∼135 kDa) were revealed by using specific antibodies of phosphor-eNOS and total eNOS. (**C**): The quantification from the blots in (A) and (B) was performed by a densitometer. Data are expressed as × Basal where the control was set as 1. Mean±SEM, *n = 3.* * p<0.05, ** p<0.01.

The Akt kinase is one of the major regulators for acute NO production [4]. In HUVECs, FSS increased Akt S473 phosphorylation (at ∼60 kDa) by over 2-fold **(** **Figure 6A and 6C** **)**. VEGF, as a positive control, produced similar effects. To confirm this positive role, the PKB/Akt specific inhibitor, triciribine [22], [23], was found to reverse the FSS-induced phosphorylation **(** **Figure 6A and 6C** **)**. Similarly, the phosphorylation of eNOS at S1177 (at ∼135 kDa) was induced by FSS, or VEGF, to over 2-fold. Triciribine also inhibited FSS-stimulated, or VEGF-stimulated, eNOS phosphorylation at S1177 without affecting the total eNOS (∼135 kDa) **(** **Figure 6B and 6C** **)**. These results suggested that the FSS-induced eNOS phosphorylation was likely to be mediated through Akt/PKB signaling pathway.

### FSS Increases Intracellular Ca^2+^ in HUVECs

**Figure 7:**
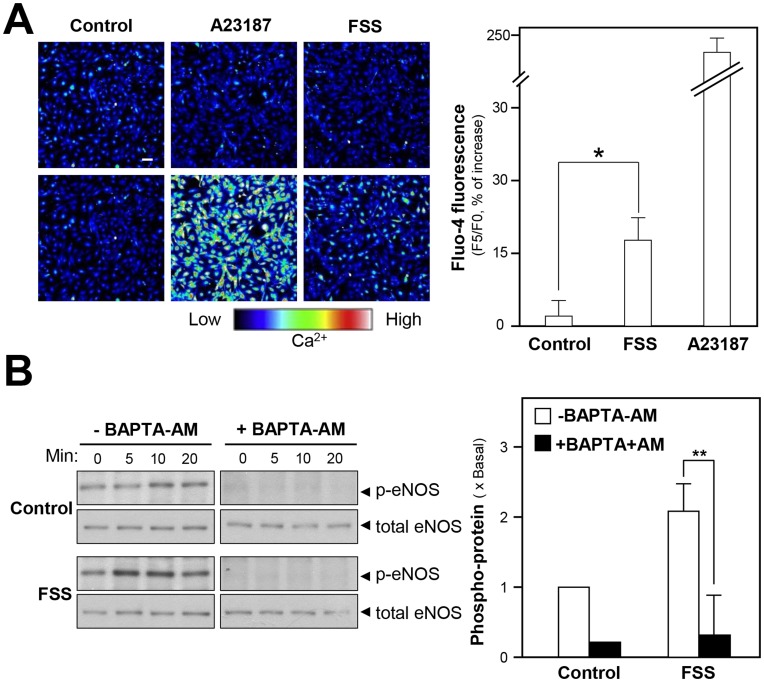
Role of intracellular Ca^2+^ in FSS-stimulated NO production. (**A**): Cultured HUVECs were labeled with fluorescent Ca^2+^ indicator Fluo-4 AM for 30 min. Then, the cells were washed with 1× NPSS (pH = 7.4), and then fluorimetric measurements were performed after the treatment of FSS (1 mg/ml), A23187 (1 µM, positive control) or control (without drug treatment). Bar = 50 µm. The photos were taken by using laser confocal fluorescent microscopy (left panel), which the fluorescence intensity excited at 488 nm and emitted at 525 nm. Quantitation of fluorescent was calculated from the labeled HUVECs (right panel). Values are expressed as the percentage of increase in the intensity of fluorescent, as compared to control cultures in time 0 sec. * p<0.05. (**B**): Cultured HUVECs were pre-treated with BAPTA-AM (5 µM) for 3 hours before the application of FSS (1 mg/ml) or control (without drug treatment) for different time points. Phospho-eNOS (∼135 kDa) and total eNOS (∼135 kDa) were revealed by using specific antibodies. Data are expressed as × Basal where the control was set as 1. Mean±SEM, *n* = 3, ** p<0.01.

Calmodulin kinase II is playing an important role in rapid activation of eNOS [13]. The increase of cytoplasmic Ca^2+^ activates calmodulin, which then binds to the calmodulin-binding domain in eNOS leading to the activation of calmodulin kinase II [24]; the activated calmodulin kinase II phosphorylates eNOS on S1177. Fluo-4 AM, a Ca^2+^ indicator, was used to test whether the FSS-induced Ca^2+^ increase could regulate the phosphorylation of eNOS. The application of FSS alone to HUVECs increased the level of Ca^2+^ by about ∼20% **(** **Figure 7A** **)**. In addition, the pre-treatment of a Ca^2+^ chelator BAPTA-AM inhibited completely the FSS-induced eNOS phosphorylation **(** **Figure 7B** **)**. These results therefore suggested that the FSS-induced eNOS phosphorylation could be partially explained by an increased intracellular Ca^2+^ levels in HUVECs.

## Discussion

Although FSS is widely used to treat vascular diseases, such as cerebral apoplexy, cerebral thrombosis, and myocardial infarction in TCM clinic in China, its pharmacological properties and cellular mechanisms of its actions are incompletely understood. We have recently started examining both chemical and biological properties of FSS [1], [2]. Functionally, FSS markedly induced the differentiation of red blood cell precursor K562 cells and stimulated the production of hemoglobin in K562 cells [2]. These new findings support the traditionally described “nourishing blood” function of FSS. In line with this blood producing function, the application of FSS onto Hep3B human hepatocellular carcinoma cells induced the expression of EPO, a key regulator for erythropoiesis. The induction of EPO expression was accompanied by an increased amount of hypoxia-inducible factor-1α [1].

We previously reported that FSS possessed the ability to inhibit the ADP-induced platelet aggregation [2], suggesting that FSS is able to facilitate blood flow. In the present study, we extended the “promoting blood circulation” property of FSS to vascular function. Here, we show for the first time that FSS effectively protects endothelial function against homocysteine insult. This benefit is likely associated with the suppression of ROS generation. Less than 60 min of exposure to high concentration of homocysteine, the ROS generation could also be mediated by NADPH oxidase family; however, which has not been addressed here. In addition, homocysteine, in aortic rings, impairs endothelium-dependent relaxations via reducing NO bioavailability [25]. Further studies using two assay methods, Griess reaction and NO-sensitive fluorescence microscopy, confirmed that FSS was capable of stimulating NO formation in human endothelial cells via increasing eNOS activity, reflected by significantly elevated the phosphorylation of eNOS. The increased eNOS activity was likely to be mediated by the increased phosphorylation of Akt kinase, an eNOS upstream regulator, and intracellular Ca^2+^ concentration in endothelial cells because both Akt inhibitor triciribine and Ca^2+^ chelator BAPTA-AM inhibited the FSS-stimulated eNOS phosphorylation.

FSS decoction is composed of two herbs: CR and ASR, and the constituents in ASR and CR are quite similar, including alkylphthalides (e.g. Z-ligustilide and senkyunolide A), phthalide dimers (e.g. levistolide A and tokinolide B), phenolic constituents (e.g. ferulic acid and coniferyl ferulate) [26]. FSS is the best known paired-herbs in the usage of Chinese medicine. Amongst all the TCM prescriptions, 43 popular paired-herbs are recorded, e.g. Ginseng Radix (root of *Panax* ginseng) and Astragali Radix (root of *Astragalus membranaceus* var. *mongholicus* or *Astragalus membranaceus*), Citri Reticulatae Pericarpium (fruit peel of *Citrus reticulata*) and Pinelliae Rhizoma (rhizome of *Pinellia ternata*), Zingiberis Recens Rhizoma (rhizome of *Zingiber officinale*) and Pinelliae Rhizoma are frequently used together. The making up of FSS having these two chemically similar herbs is not known. According to the basic theory of TCM prescription, the purpose of TCM paired-herbs is to produce synergistic effects aiming to enhance therapeutic efficacy and/or to minimize toxicity and adverse effects. For FSS, the reduction of side effects might be a possible main reason of using ASR and CR together [1], [2]. In ancient China, the original usage of FSS was for abortion, which could lead to a drastic loss of blood; this effect can be triggered by CR. Having ASR in FSS is likely to compensate for the depressed blood functions related to CR.

Although the active components in FSS have not been revealed in the present study, Z-Ligustilide and ferulic acid are considered to be active constituents in both ASR and CR. Ferulic acid possesses a vasodilator and hypotensive effect in spontaneously hypertensive rats [27]. In addition, the sodium salt of ferulic acid induced the NO production [28]: this induction was possibly mediated by increased Ca^2+^ influx in endothelial cells. In HVUECs, the application of ferulic acid protected the cells against radiation-induced oxidative stress [29]. Furthermore, ferulic acid reduced the generation of NADPH-dependent production of superoxide anion [30]. The aforementioned different lines of evidence suggest the vascular benefits of FSS. Although FSS can trigger beneficial pharmacological actions, the responsible active compounds have never been identified. This is a conundrum not only for FSS but also for the vast majority of TCM prescriptions.

The herbal decoction generally has multiple components acting on multiple targets. Combining appropriate experimental models such as blood vessels and endothelial cells in the present study with the theory of TCM, the mechanisms of actions of herbal decoction can be gradually revealed. On the other hand, better understanding of molecular pathways involved in TCM actions shall provide useful information relevant to therapeutic applications of TCM. In summary, the present study elucidates that the cellular and molecular mechanisms of FSS in blood vessels and in human endothelial cells. The primary vascular benefit is attributed to FSS’s ability to stimulate the eNOS-derived NO production via: (i) PKB/Akt signaling pathway; (ii) increased intracellular Ca^2+^; and (iii) reduced ROS generation. Our experimental findings shall enhance the prospects of using FSS in combating against certain forms of cardiovascular diseases in oriental populations.

## Materials and Methods

### Plant Materials and Preparation of Fo Shou San

Fresh roots were obtained from China from September to October of 2008, listed as follows: 2-year-old ASR (roots of *Angelica sinensis* (Oliv.) Diels.) from Minxian of Gansu province and 3-year-old CR (rhizomes of *Ligusticum chuanxiong* Hort.) from Guanxian of Sichuan province. These areas were known to produce the best quality of ASR and CR, respectively [26]. The herbs were authenticated by one of the authors, Dr. Tina Dong. The corresponding vouchers as forms of whole plants, voucher # 08-11-1 for CR voucher # 08-9-1 for ASR, were deposited in Center for Chinese Medicine, The Hong Kong University of Science and Technology. The raw materials were purchased from medicinal materials market. No specific permissions were required for the locations or activities during the collection of the raw material, the location is also not privately-owned or protected, and our studies did not involve endangered or protected species. In the preparation of FSS, the amounts of crude drugs of CR and ASR were weighed according to the weight ratio of 2∶3 and then mixed well by vortexing. The herbal mixture was boiled in 8 volumes of water (v/w) for 1 hour and extracted twice. For the second extraction of FSS, the residue from the first extraction was filtered before the process. The extracts were dried by lyophilization and stored at 4°C.

### Quantitative Analysis

Ferulic acid was purchased from Sigma (St. Louis, MO). Ligustilide (Z-isoform) was kindly provided by Prof. Pengfei Tu from Peking University; their purities, confirmed by HPLC, were higher than 99.0%. AR- and High Performance Liquid Chromatography (HPLC)-grade reagents were from Merck (Darmstadt, Germany). A Waters (Milford, MA) HPLC system consisting of a 600 pump, a 717 auto-sampler, and a UV/VIS Photodiode Array 2996 Detector was used for all analyses. Chromatographic separations were carried out on a Phenomenex C_18_ column (particle size 5 µm, 4.60 mm×250 mm) with 1% acetate acid in water (as solvent A): acetonitrile (as solvent B) in the mobile phase at a flow rate of 1.0 ml/min at room temperature. A gradient elution was applied from 0 to 81% of solvent A starting from 0 to 18 min, from 81% to 0 of solvent A starting from 18 to 60 min, and 0 of A starting from 60 to 75 min. Samples were filtered through a 0.45 µm Millipore syringe filter unit. Ten µl samples were injected for HPLC analysis. For the calibration of ferulic acid and Z-ligustilide, the standards were weighed and dissolved in methanol to give serial concentrations from 1 to 100 mg/l, and two injections onto HPLC were performed for each dilution. The concentrations of these compounds in the samples were calculated according to the regression parameters derived from the standard curve (**Table S1**).

### Artery Preparation

Male Sprague–Dawley rats (∼250–300 g) were sacrificed by cervical dislocation and bled. The thoracic aorta was excised. After surrounding connective tissue had been carefully cleaned off, four 3-mm-wide and 2-mm-long ring segments were prepared from each aorta. The rings were suspended between two stainless wire hooks in a 10-ml organ bath [31]. The upper wire was connected to a force–displacement transducer (Grass Instruments, Rockland, MA), and the lower one was fixed at the bottom of the organ bath. The organ bath was filled with Krebs solution of the following composition: 119 mM NaCl, 4.7 mM KCl, 25 mM NaHCO_3_, 2.5 mM CaCl_2_, 1 mM MgCl_2_, 1.2 mM KH_2_PO_4_, and 11 mM D-glucose. The bathing solution was gassed with 95% O_2_–5% CO_2_ at 37°C (pH = 7.4). The rings were placed under an optimal basal tone of 20 mN, determined from previous length–tension experiments. Changes in isometric tension were measured with a Grass force transducer and stored on MacLab software (Version 3.0, AD Instruments, Colorado Spring, CO) for later data analysis. Twenty minutes after mounting in organ baths, the rings were first contracted with 0.5 µM phenylephrine to test the contractility and then relaxed by the application of ACh. They were rinsed several times until baseline tone was restored. The rings were thereafter allowed to equilibrate for 60 min. Baseline tone was re-adjusted to 20 mN when necessary. Each set of experiments was performed on rings prepared from different rats. The use of laboratory animals was approved by the Animal Research Ethical Committee of the Chinese University of Hong Kong.

### HUVEC Culture

HUVEC cell line was obtained from Lonza (San Diego, CA) and cultured on 0.2% gelatin-coated tissue culture plate in Lonza Endothelial Growth Medium (EGM) Bulletkit in a humidified incubator at 37°C with 95% air, 5% CO_2_. HUVECs between passages 3 and 8 were used in these studies as to ensure the genetic stability of the culture. All culture reagents were purchased from Lonza.

### Nitric Oxide Colorimetric Assay

Cultured HUVECs were seeded onto a 96-well plate at a density of 2,000 cells/well in 0.1 ml of EGM Bulletkit medium. The medium was replaced by 0.1 ml of serum and growth factor free medium containing FSS every day. The concentrations of NO in the culture medium were measured with the NO Detection Kit (Biovision, Mountain View, CA) according to the manufacturer’s instructions. NO is rapidly oxidized to nitrite and nitrate, which are the mean to determine the NO production. The amount of nitrate was determined by converting it to nitrite, followed by the colorimetric determination of the total concentration of nitrite as a colored azo dye product of the Griess reaction that absorbed visible light at 540 nm using a microplate reader [32].

### Laser Confocal Fluorescent Microscopy

Fluorimetric measurements were performed on cultured HUVECs using an Olympus Fluoview FV1000 laser scanning confocal system (Olympus America Inc., Melville, NY) mounted on an inverted I ×81 Olympus microscope, equipped with a 10× objective (NA 0.5). Intracellular NO production was monitored using fluorescent NO indicator 4-amino-5-methylamino-20, 70-difluorofluorescein diacetate (DAF-FM DA, Invitrogen, Grand Island, NY). Cultured HUVECs seeded on glass coverslips were incubated for 30 min at room temperature in normal physiological saline solution (NPSS) containing 1 µM DAF-FM DA. The amount of NO was evaluated by measuring the fluorescence intensity excited at 495 nm and emitted at 515 nm. Changes in intracellular NO production were displayed as a ratio of fluorescent intensity at any time relative to control at time 0 (Fn/F0) [33]. To measure the formation of reactive oxygen species (ROS), cultured HUVECs were labeled by 100 µM dichlorofluorescein diacetate (DCFH-DA) in NPSS buffer. The intracellular ROS generation of cells can be investigated using DCFH-DA by converting DCFH to DCF through the action of peroxide rated by the presence of peroxidase. The fluorescence intensity of DCF excited at 505 nm and emitted at 535 nm [34]. NPSS contained 140 mM NaCl, 5 mM KCl, 1 mM CaCl_2_, 1 mM MgCl_2_, 10 mM glucose, and 5 mM HEPES (pH 7.4). To measure the Ca^2+^ influx, cultured HUVECs were labeled with 5 µM of Fluo-4 AM in NPSS buffer. The Ca^2+^ influx could be indicated by the fluorescence intensity excited at 488 nm and emitted at 525 nm [35].

### Western Blot Analysis

Cultured HUVECs were treated with different drugs, and then which were collected to perform western blot analysis. For the determination of eNOS and Akt proteins, the cell lysate was collected in lysis buffer (0.125 M Tris-HCl, pH 6.8, 4% SDS, 20% glycerol, and 2% 2-mercaptoethanol) and then denatured at 100°C for 10 min, which was then subjected to 8% SDS-PAGE and western blotting. The phospho-eNOS S1177, total eNOS (∼135 kDa), phospho-AKT S473 and total Akt (∼60 kDa) were identified by specific antibodies. The anti-eNOS, anti-phospho-eNOS S1177, anti-Akt, anti-phospho-Akt S473 antibodies (1∶1,000; Cell Signaling, Danvers, MA) and HRP-conjugated anti-rabbit secondary antibody (1∶5,000, Cell signaling) were employed here. The eNOS dimers were assayed using low-temperature SDS-PAGE under non-reducing conditions, as described previously [36], [37], [38]. After treatment, the cell lysate was collected using lysis buffer (without 2-mercaptoethanol), in order to test the possibility of dimer dissociation by reduction of disulfide bridges of eNOS protein. Samples were subjected to SDS-PAGE on 6% gels, gels and buffers were kept in an ice bath at 4°C. The membranes were incubated with a monoclonal antibody against total eNOS (1∶1,000) overnight at 4°C. The immune complexes were visualized using the enhanced chemiluminescence (ECL) method (GE Healthcare, Piscataway, NJ). The intensities of the bands in the control and different samples, run on the same gel and under strictly standardized ECL conditions, were compared on an image analyzer, using a calibration plot constructed from a parallel gel with serial dilutions of one of the samples.

### Measurement of ROS Production by Lucigenin-enhanced Chemiluminescence

The generation of O_2_•^−^ was measured using a lucigenin (Bis-*N*-methylacridinium Nitrate, Sigma)-enhanced chemiluminescence technique [39], [40], [41]. Briefly, HUVECs was seed in culture flask, and pre-incubated with different drugs. Cells were then detached using 0.25% trypsin-EDTA (1 mM), and washed with 1× PBS. The cells were resuspended at 10^6^/ml in HEPEs buffer containing (mM): NaCl 140, KCl 5, MgCl_2_ 0.8, CaCl_2_ 1.8, Na_2_HPO_4_ 1, HEPEs 25 and 1% glucose, pH 7.4). Cells were distributed at 5×10^4^ per well on a 96-well white plate. Before recording the chemiluminescence, NADPH (100 µM) and lucigenin (25 µM) was added via the auto-dispenser. H_2_O_2_ was used as an assay control. The light emission between O_2_•^−^ and lucigenin was detected and quantified by FLUOstar Optima (BMG Labtech) over 20 min. This time point was determined to be optimized [41].

### Protein Assay

The concentration of protein was determined following the instructions of the manufacturer. The analysis was done on 96-well microtiter plate. Essentially, one part dye reagent concentrate was diluted with 4 parts of double distilled water before use. Five dilutions of BSA standard (0.1–0.5 µg/ml) were used for the test. The samples were assayed in triplicate readings. Ten µl of each standard and sample solution were added with 0.2 ml of diluted dye reagent into separate wells and mixed well. After 5-min incubation at room temperature, the absorbance at 595 nm was taken. The concentration of protein was determined from the standard curve.

### Statistical Analysis

All data were analyzed by one-way ANOVA (analysis of F-test, q-test and t-test) using the SPSS version 11 statistical analysis program. Statistical significance was considered as ** for p<0.01 and *** for p<0.001.

## Supporting Information

Figure S1 **(A):** The structures and HPLC chromatography of the chemical markers, ferulic acid senkyunolide I, senkyunolide H and Z-ligustilide, were shown. **(B):** The HPLC analysis (at an absorbance of 280 nm) was performed to illustrate the standard chemical markers, ferulic acid, senkyunolide I, senkyunolide H and Z-ligustilide. Results are Means ± SEM, *n* = 3.
(TIF)

Figure S2 **Rat aortic ring was isolated.** The tension responsible for the vascular relaxation and constriction was tested after the treatment of different drugs as in **Figure 1**. The endothelium dysfunction was induced by homocysteine (300 µM, a ROS inducer) for 60 min. The ring was suspended between two stainless steelwires in a 10-ml chamber on a Multi Myograph, which was used to measure the tension. Concentration-response curves for acetylcholine (ACh from 0.01 to 10 µM) in the absence (control), or in the presence of homocysteine, or the co-treatment of homocysteine with FSS (3 mg/ml), tempol (1 µM, a ROS scavenger), apocynin (1 µM, an inhibitor of NADPH oxidase), catalase (1000 U/ml), oxypurinol (100 µM, a xanthine oxidase inhibitor) were tested. Values are expressed as percentage of relaxation as comparing to the control resting tension. Mean ± SEM, *n* = 4.
(TIF)

Figure S3 **Rat aortic ring was isolated (as in** **Figure 1** **) and scratch to get rid of the intact of endothelium.** The ring was suspended between two stainless steelwires in a 10-ml chamber on a Multi Myograph, which was used to measure the tension. A concentration-response curve for FSS (0.1–10 mg/ml) was tested.
(TIF)

Figure S4 **Rat aortic ring was isolated.** The tension responsible for the vascular relaxation and constriction was tested after the treatment of different drugs as in **Figure 1**. The endothelium dysfunction was induced by homocysteine (300 µM, a ROS inducer) for 60 min. The ring was suspended between two stainless steelwires in a 10-ml chamber on a Multi Myograph, which was used to measure the tension. Acetylcholine (ACh from 0.01 to 10 µM; see **Figure 1**) was added (as indicated) in the absence of homocysteine (control). The internal control of rat aortic ring relaxation to homocysteine alone (300 µM), or the co-treatment of homocysteine with FSS (3 mg/ml), tempol (1 µM, a ROS scavenger), apocynin (1 µM, an inhibitor of NADPH oxidase) and catalase (1000 U/ml) were tested without being induced by the application of ACh.
(TIF)

Figure S5 **Cultured HUVECs were seeded in 12-well plates.** The cultures were serum starved for 3 hours before the pre-treatment with homocysteine (300 µM) for another 1 hour. The cells were then treated with FSS (1 mg/ml), or VEGF (20 ng/ml, positive control), or control (without drug treatment), at different time points. Total and phosphorylated eNOS was revealed by using specific antibodies, as in **Figure 6**.
(TIF)

Figure S6 **Cultured HUVECs were treated with FSS (1 mg/ml) for 24 hours and cell viability was tested using MTT assay.** Data are expressed as percentage of increase compared to control (without FSS treatment). Results are Means ± SEM, *n* = 4.
(TIF)

Figure S7 **Rat aortas were isolated (as in** **Figure 1** **) and cut into spiral strips (2 mm×1 cm), which were then mounted in 5 ml myograph bath containing Krebs solution and gassed with 95% O_2_, 5% CO_2_ in 37°C.** After exposure to different drugs as indicated, the tissues were quickly frozen in liquid nitrogen, and cGMP levels were assayed as described in Direct cGMP ELISA kit (Enzo Life Sciences, Farmingdale, NY). The aortas were treated with Phe (0.5 µM) first for about 3 min, and then add FSS (3 mg/ml). For the pre-treatment, L-NAME (100 µM) was added for 45 min. Values of cGMP are expressed as X Basal, where the control was set as 1. Mean ± SEM, *n* = 4.
(TIF)

Table S1 **Calibration curves the chemical markers.** ^a^ These calibration curves were constructed by plotting the peak area versus the concentration of each analyte. Each calibration curve was derived from four data points, *n = *3, and the SD was <5% of the Mean.
(TIF)
