# Supplementary figures and images for: Fo Shou San, an Ancient Chinese Herbal Decoction, Protects Endothelial Function through Increasing Endothelial Nitric Oxide Synthase Activity
Source: PLoS One. 2012 Dec 21;7(12):e51670. doi: 10.1371/journal.pone.0051670 (PMC3528755; doi:10.1371/journal.pone.0051670)

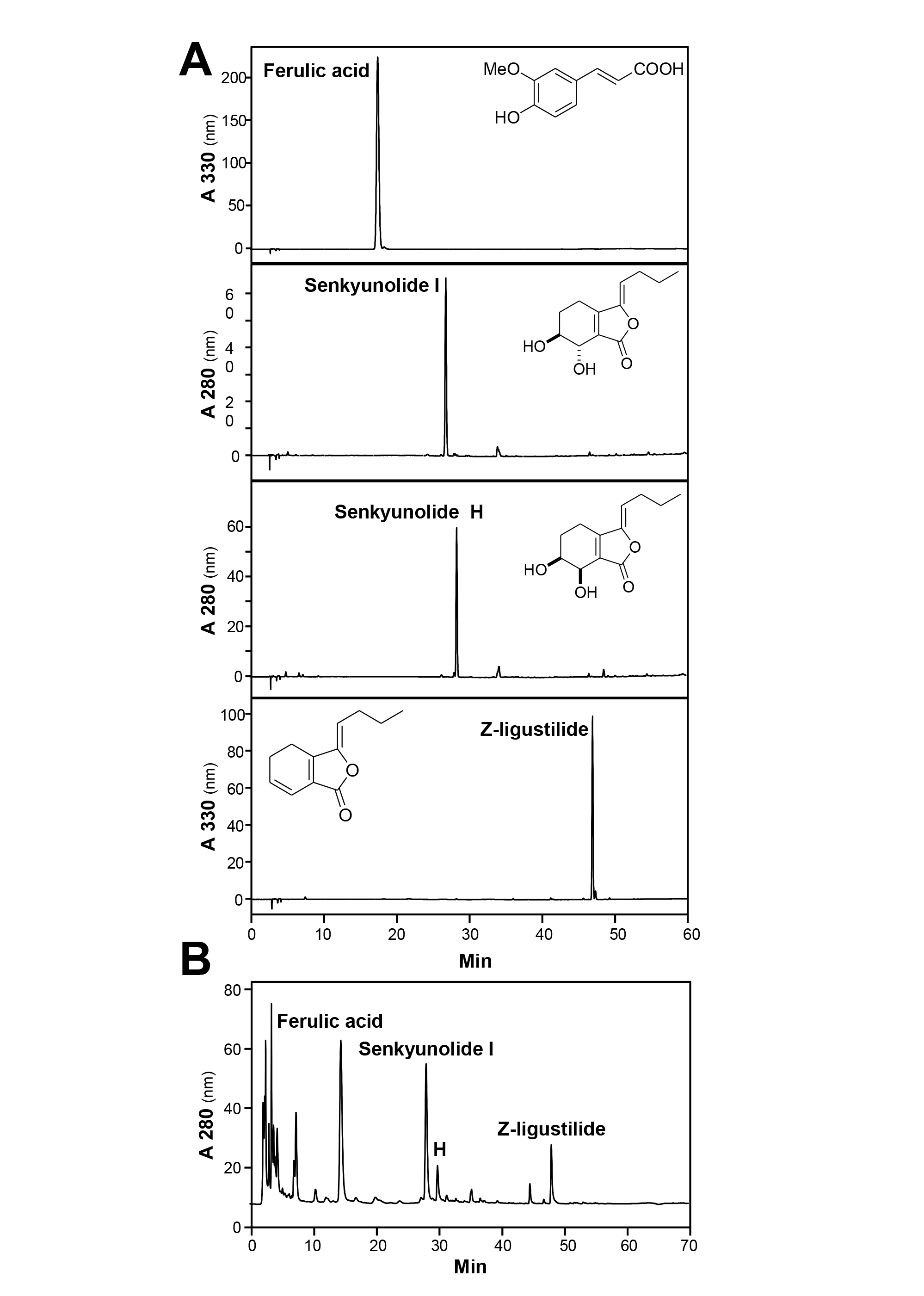

Supplement: Figure S1 — (A): The structures and HPLC chromatography of the chemical markers, ferulic acid senkyunolide I, senkyunolide H and Z-ligustilide, were shown. (B): The HPLC analysis (at an absorbance of 280 nm) was performed to illustrate the standard chemical markers, ferulic acid, senkyunolide I, senkyunolide H and Z-ligustilide. Results are Means ± SEM, n = 3. (TIF) [file pone.0051670.s001.tif]

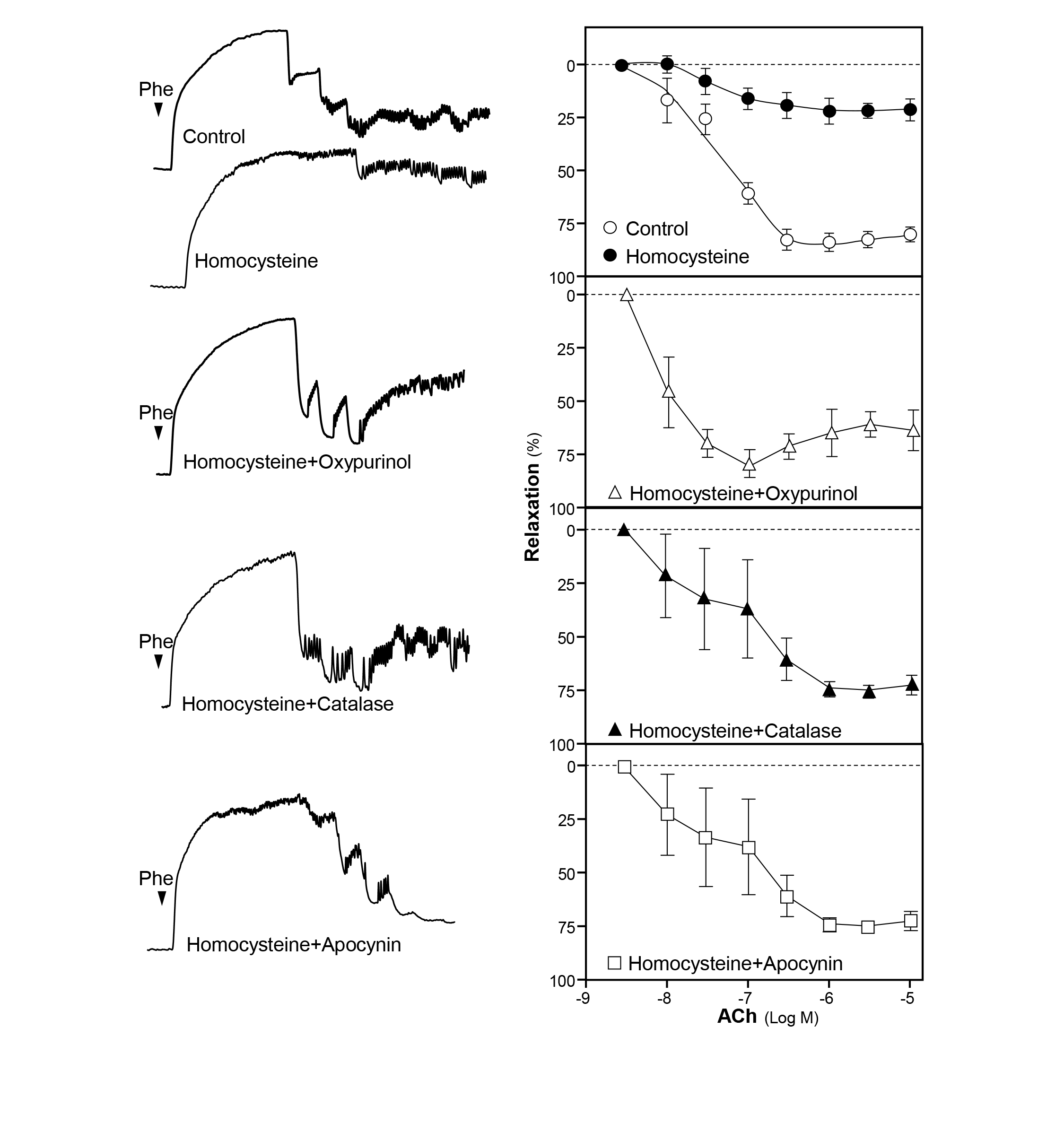

Supplement: Figure S2 — Rat aortic ring was isolated. The tension responsible for the vascular relaxation and constriction was tested after the treatment of different drugs as in Figure 1 . The endothelium dysfunction was induced by homocysteine (300 µM, a ROS inducer) for 60 min. The ring was suspended between two stainless steelwires in a 10-ml chamber on a Multi Myograph, which was used to measure the tension. Concentration-response curves for acetylcholine (ACh from 0.01 to 10 µM) in the absence (control), or in the presence of homocysteine, or the co-treatment of homocysteine with FSS (3 mg/ml), tempol (1 µM, a ROS scavenger), apocynin (1 µM, an inhibitor of NADPH oxidase), catalase (1000 U/ml), oxypurinol (100 µM, a xanthine oxidase inhibitor) were tested. Values are expressed as percentage of relaxation as comparing to the control resting tension. Mean ± SEM, n = 4. (TIF) [file pone.0051670.s002.tif]

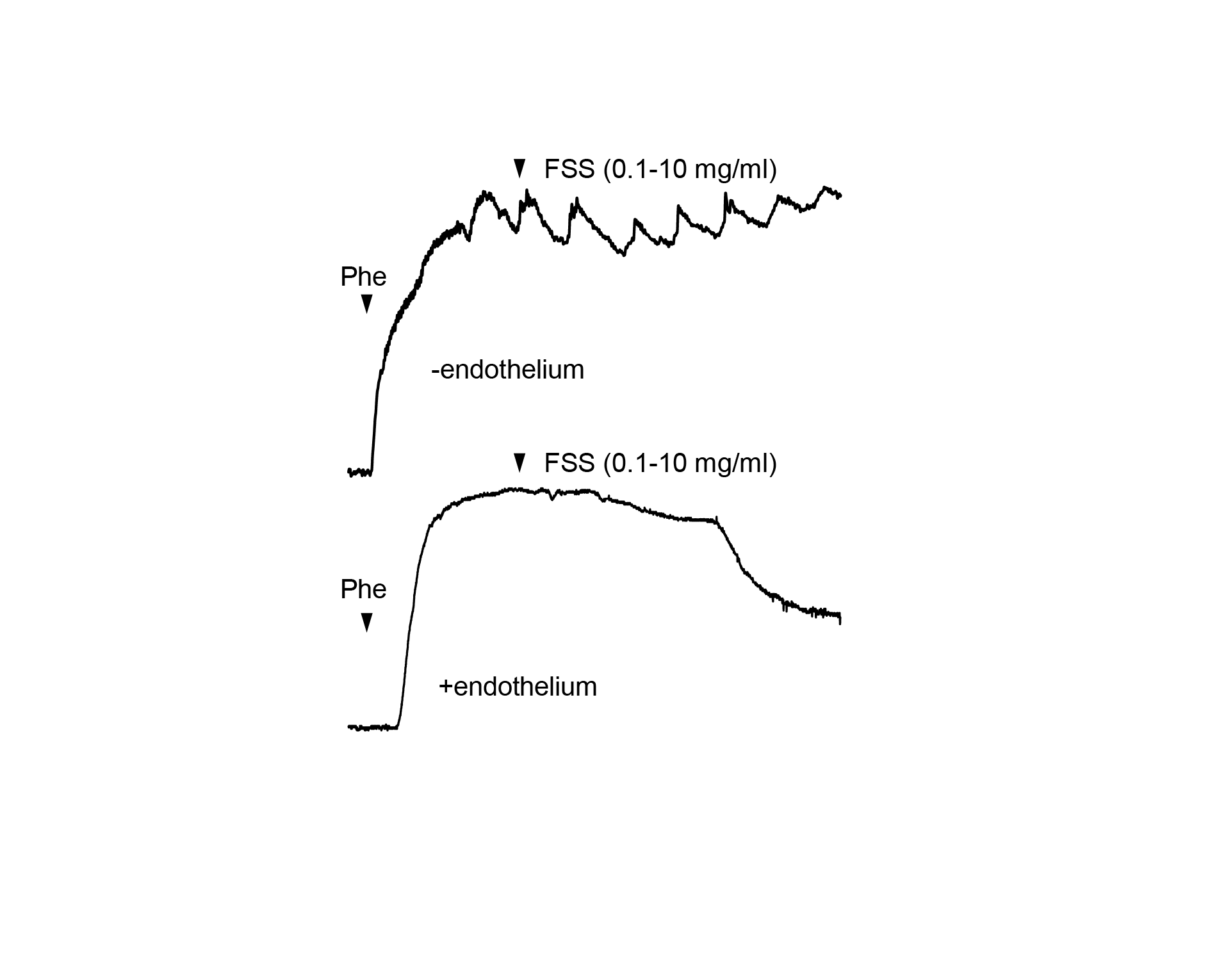

Supplement: Figure S3 — Rat aortic ring was isolated (as in Figure 1 ) and scratch to get rid of the intact of endothelium. The ring was suspended between two stainless steelwires in a 10-ml chamber on a Multi Myograph, which was used to measure the tension. A concentration-response curve for FSS (0.1–10 mg/ml) was tested. (TIF) [file pone.0051670.s003.tif]

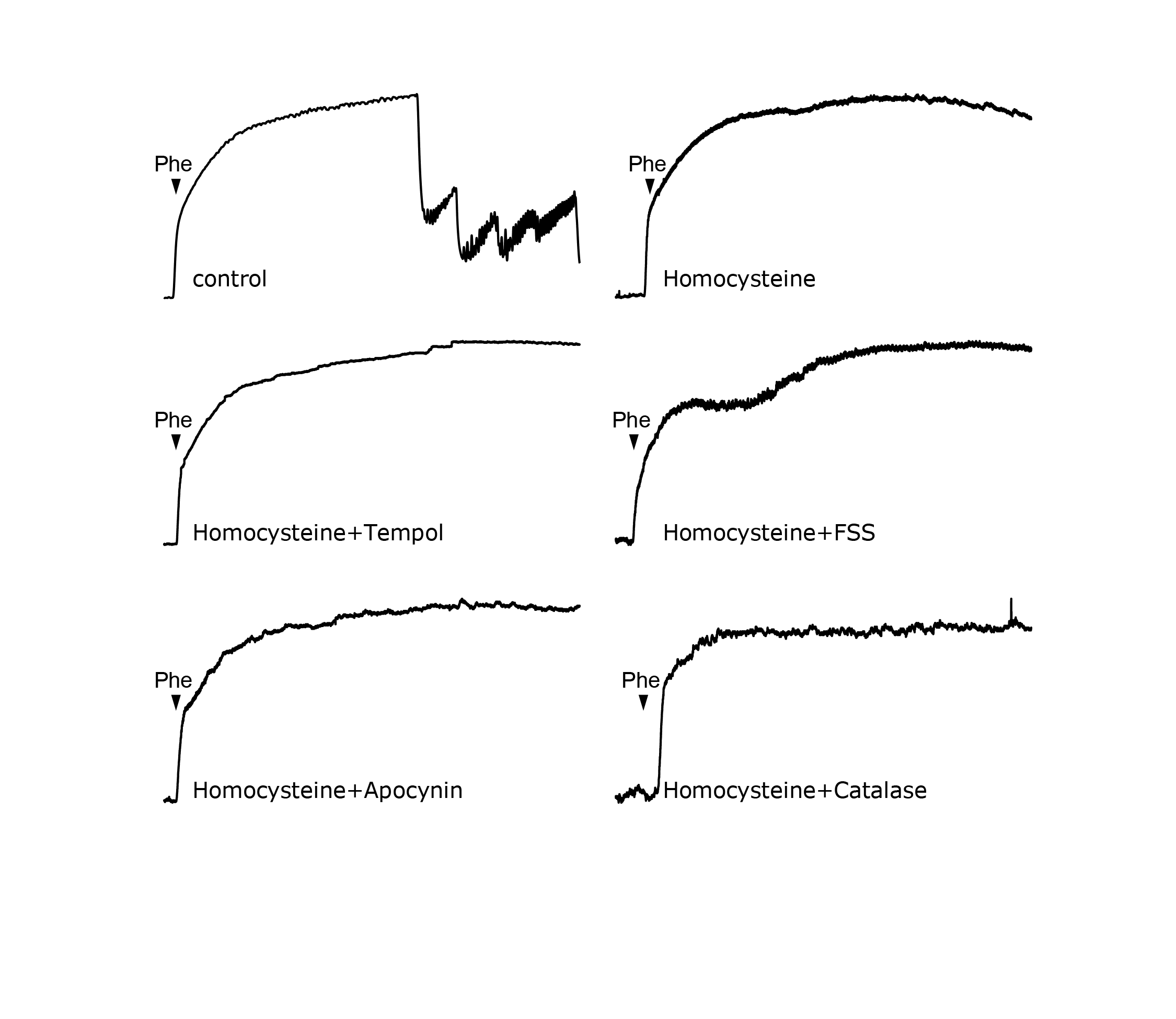

Supplement: Figure S4 — Rat aortic ring was isolated. The tension responsible for the vascular relaxation and constriction was tested after the treatment of different drugs as in Figure 1 . The endothelium dysfunction was induced by homocysteine (300 µM, a ROS inducer) for 60 min. The ring was suspended between two stainless steelwires in a 10-ml chamber on a Multi Myograph, which was used to measure the tension. Acetylcholine (ACh from 0.01 to 10 µM; see Figure 1 ) was added (as indicated) in the absence of homocysteine (control). The internal control of rat aortic ring relaxation to homocysteine alone (300 µM), or the co-treatment of homocysteine with FSS (3 mg/ml), tempol (1 µM, a ROS scavenger), apocynin (1 µM, an inhibitor of NADPH oxidase) and catalase (1000 U/ml) were tested without being induced by the application of ACh. (TIF) [file pone.0051670.s004.tif]

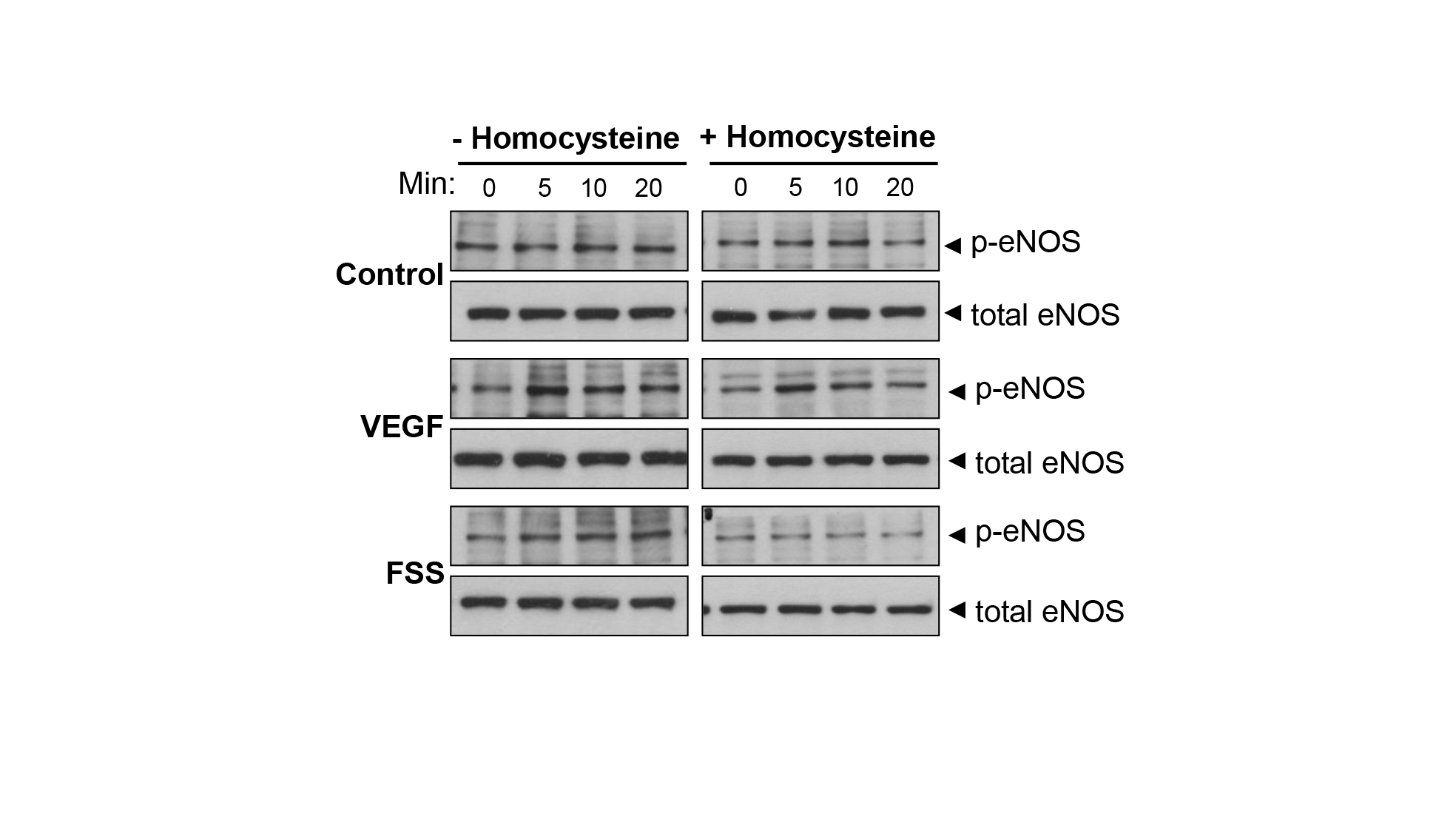

Supplement: Figure S5 — Cultured HUVECs were seeded in 12-well plates. The cultures were serum starved for 3 hours before the pre-treatment with homocysteine (300 µM) for another 1 hour. The cells were then treated with FSS (1 mg/ml), or VEGF (20 ng/ml, positive control), or control (without drug treatment), at different time points. Total and phosphorylated eNOS was revealed by using specific antibodies, as in Figure 6 . (TIF) [file pone.0051670.s005.tif]

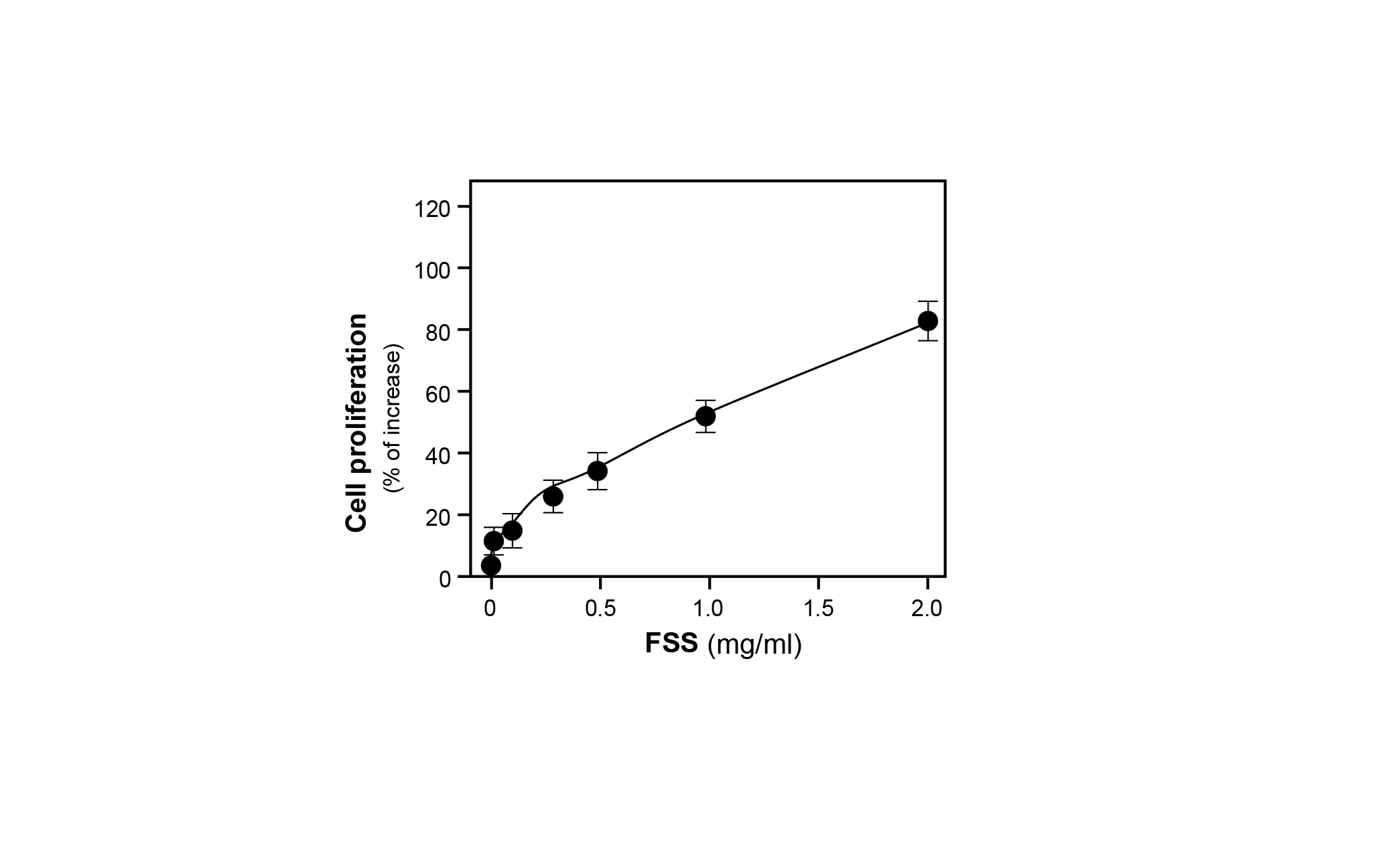

Supplement: Figure S6 — Cultured HUVECs were treated with FSS (1 mg/ml) for 24 hours and cell viability was tested using MTT assay. Data are expressed as percentage of increase compared to control (without FSS treatment). Results are Means ± SEM, n = 4. (TIF) [file pone.0051670.s006.tif]

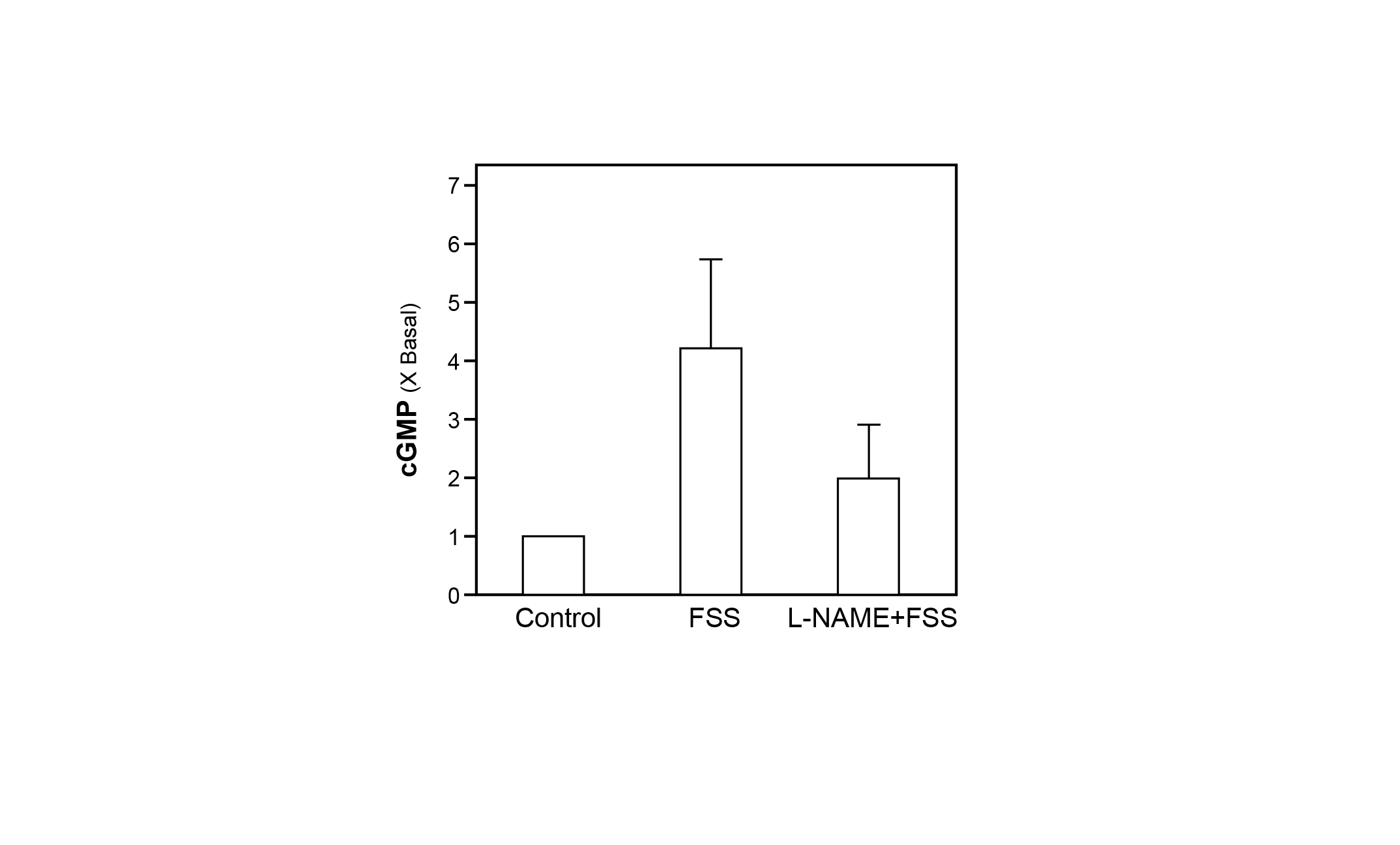

Supplement: Figure S7 — Rat aortas were isolated (as in Figure 1 ) and cut into spiral strips (2 mm×1 cm), which were then mounted in 5 ml myograph bath containing Krebs solution and gassed with 95% O2, 5% CO2 in 37°C. After exposure to different drugs as indicated, the tissues were quickly frozen in liquid nitrogen, and cGMP levels were assayed as described in Direct cGMP ELISA kit (Enzo Life Sciences, Farmingdale, NY). The aortas were treated with Phe (0.5 µM) first for about 3 min, and then add FSS (3 mg/ml). For the pre-treatment, L-NAME (100 µM) was added for 45 min. Values of cGMP are expressed as X Basal, where the control was set as 1. Mean ± SEM, n = 4. (TIF) [file pone.0051670.s007.tif]

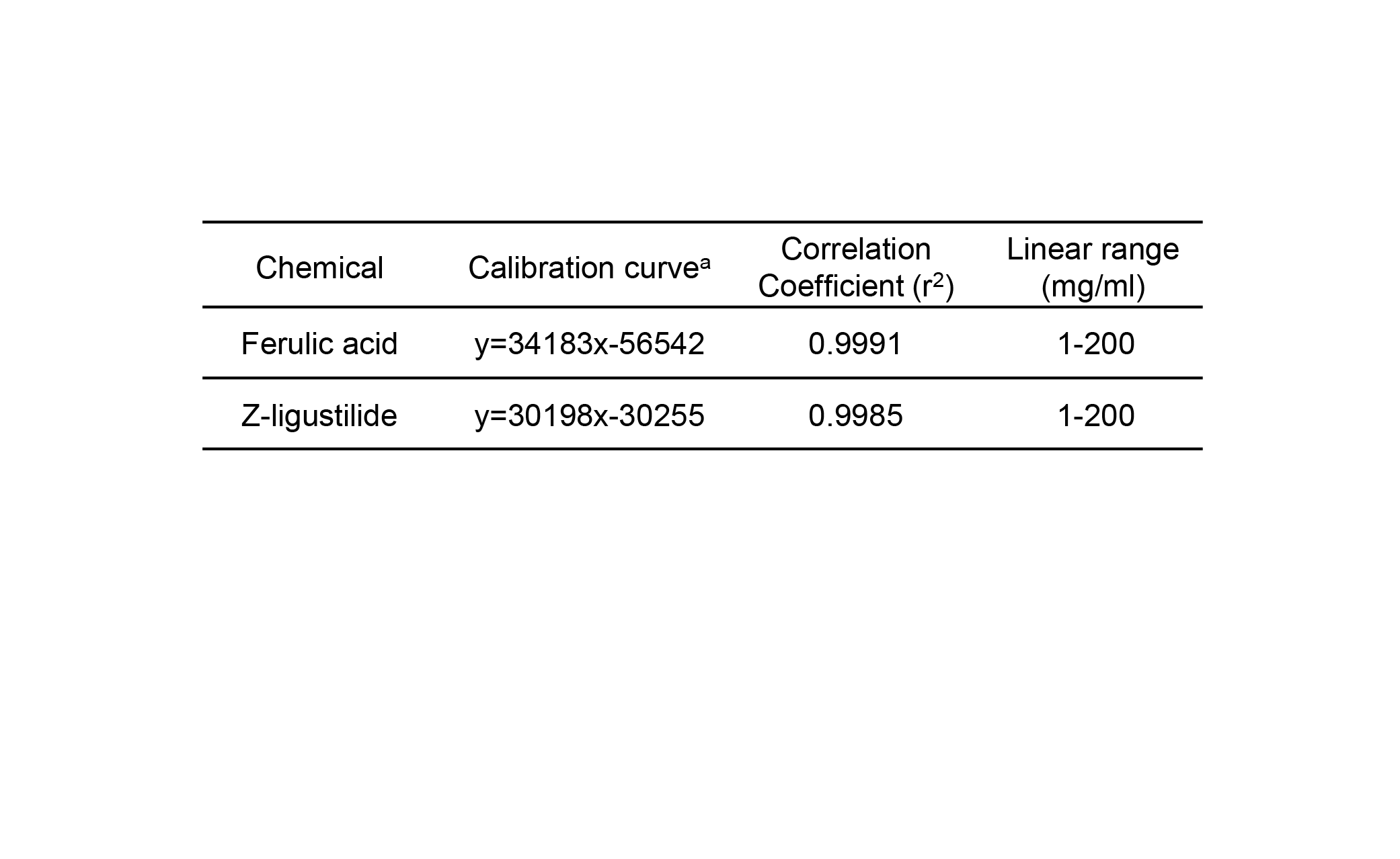

Supplement: Table S1 — Calibration curves the chemical markers. a These calibration curves were constructed by plotting the peak area versus the concentration of each analyte. Each calibration curve was derived from four data points, n = 3, and the SD was <5% of the Mean. (TIF) [file pone.0051670.s008.tif]
